# A Fast and On-Machine Measuring System Using the Laser Displacement Sensor for the Contour Parameters of the Drill Pipe Thread

**DOI:** 10.3390/s18041192

**Published:** 2018-04-13

**Authors:** Zhixu Dong, Xingwei Sun, Changzheng Chen, Mengnan Sun

**Affiliations:** 1 School of Mechanical Engineering, Shenyang University of Technology, Shenyang 110870, China; sunxw@sut.edu.cn (X.S.); czchen@sut.edu.cn (C.C.); 2 School of Mechanical Engineering & Automation, Northeastern University, Shenyang 110819, China; 1410112@stu.neu.edu.cn

**Keywords:** drill pipe thread, on-machine measurement, laser displacement sensor, wavelet threshold denoising

## Abstract

The inconvenient loading and unloading of a long and heavy drill pipe gives rise to the difficulty in measuring the contour parameters of its threads at both ends. To solve this problem, in this paper we take the SCK230 drill pipe thread-repairing machine tool as a carrier to design and achieve a fast and on-machine measuring system based on a laser probe. This system drives a laser displacement sensor to acquire the contour data of a certain axial section of the thread by using the servo function of a CNC machine tool. To correct the sensor’s measurement errors caused by the measuring point inclination angle, an inclination error model is built to compensate data in real time. To better suppress random error interference and ensure real contour information, a new wavelet threshold function is proposed to process data through the wavelet threshold denoising. Discrete data after denoising is segmented according to the geometrical characteristics of the drill pipe thread, and the regression model of the contour data in each section is fitted by using the method of weighted total least squares (WTLS). Then, the thread parameters are calculated in real time to judge the processing quality. Inclination error experiments show that the proposed compensation model is accurate and effective, and it can improve the data acquisition accuracy of a sensor. Simulation results indicate that the improved threshold function is of better continuity and self-adaptability, which makes sure that denoising effects are guaranteed, and, meanwhile, the complete elimination of real data distorted in random errors is avoided. Additionally, NC50 thread-testing experiments show that the proposed on-machine measuring system can complete the measurement of a 25 mm thread in 7.8 s, with a measurement accuracy of ±8 μm and repeatability limit ≤ 4 μm (high repeatability), and hence the accuracy and efficiency of measurement are both improved.

## 1. Introduction

The drill pipe thread is mainly used for the connection between drill pipes in the oil and gas drilling industry, and it can transmit the great torque required by the drilling from the wellhead to the bottom hole bit [1]. As this taper thread bears great torque and high sealing requirements, its contour parameters should be highly accurate and conform to the standards of American Petroleum Institute (API) [2]. At present, the measurement of the contour parameters of the drill pipe thread is a contact type in the following two categories: (1) One category involves the measurement of the thread standoff by wrenching the thread gauge, which is the only method to judge whether the drill pipe thread processed is qualified by each oilfield service station. Though both the on-machine and off-machine measuring methods can be carried out in the said method, we can acquire only comprehensive parameters but cannot achieve the accuracy measurement of the contour parameters of the drill pipe thread and the crest and root profile. In addition, this measurement requires hard labor and is easily affected by human factors. (2) The other involves employing the monomial parameter gauge to measure all the parameters of the drill pipe thread other than the thread angle and pitch diameter. We can conduct an off-machine, random inspection of the drill pipe thread only in the laboratory, while the measurement of parameters of the internal thread is incomplete. In recent years, many scholars have devoted themselves to research of the non-contact measurement of the thread, achieving certain results [3,4,5]. However, such research is still carried out by the off-machine measuring method. Accordingly, repeated clamping processes of a drill pipe approximately 10 m in length and 300 kg in weight cost a multitude of auxiliary time, increase the positioning error of measurement, and make it impossible to ensure the accuracy of unqualified thread after correction.

In non-contact measurement, the laser displacement sensor is widely used in the fields of reverse engineering and accuracy measurement by virtue of its advantages of high measurement accuracy, wide measurement range, easy integration with a computer to form an intelligent testing system, and ability to conduct online testing at a production site [6]. Li et al. adopted multiple laser displacement sensor arrays to realize the on-line in-situ testing of important parameters of automotive engine cabinets and connecting rods [7,8]; Wang et al. applied the laser displacement sensor to the dimension measurement of coaxial holes and then verified by experiment that the new testing method is both rapid and effective [9]; An automatic measuring system for the dimensions of the internal holes of long-stepped pipes based on the laser displacement sensor was designed [10]; Chen et al. researched a non-contact pulse automatic positioning measurement system for traditional Chinese medicine in combination with the advantages of optical measurement and computer automation [11]. However, the said research all lacks the error compensation and reconstruction of the data acquired by a laser displacement sensor, resulting in unsatisfactory measurement results. Korosec and Duhovnić proposed that the final accuracy of reverse engineering and accuracy measurement depends on the accuracy of the data acquisition by a sensor and the subsequent data reconstruction [12]. With regard to the said two aspects, some research has been carried out by scholars both at home and aboard. In terms of the data acquisition accuracy, Alam et al. researched the impact of environmental factors such as temperature, humidity, mechanical vibration, and external light on the laser measurement accuracy. Finally, Alam proposed a calibration method to improve the laser measurement accuracy and used dual non-diffracting beams as a light source to enhance the anti-noise performance of laser measurement [13]. After testing the impact of the reflectivity of 13 materials on the laser measurement accuracy, a method for evaluating the uncertainty of laser triangulation micro-measurement was proposed [14]; Rico et al. proposed such a holographic measurement technique based on the laser triangulation method that reduces the measurement error caused by reflectivity, colors, and speckle noises [15]. Yang et al. analyzed the reason for the laser beam dithering in a laser displacement sensor and then proposed a method for suppressing the dithering impact, improving the measurement accuracy of the laser displacement sensor to a certain degree [16]. Considering that the measuring point inclination angle makes a greater impact on the data acquisition accuracy of a laser displacement sensor than other factors, Lee and Shiou carried out qualitative research to analyze the mechanism of the occurrence of errors by measuring point inclination angles, acquired the law of errors caused by the object plane tilt, and finally proposed an error compensation solution [17]. Sun and Li built a quantitative inclination error compensation model based on the geometrical relationship between the incidence and receipt of the light centroid. However, there were numerous similar treatments in the modeling, resulting in larger deviations of calculation results [18]. In terms of the data reconstruction accuracy, due to being affected by the surface topography of the object measured and other factors, the point cloud data scanned by the laser displacement sensor contains random noises. Therefore, the data acquired should be denoised. The essential problem in data reconstruction is to remove the noise and maintain the details of the original information as far as possible [19]. Recently, the discrete wavelet transform (DWT) has proven to be a key technique for eliminating the noise because of its effectiveness. However, the data processing quality of DWT denoising methods is influenced by the construction of a threshold function immediately [20]. Donoho and Johnstone presented the hard and soft threshold functions that are widely used in DWT denoising [21]. However, there are two drawbacks to the said two methods: discontinuity in the hard denoising function and the constant deviation in the soft denoising function. Nasri [22], Yi [23], Gong [24], and Zhang [25], according to the characteristics of the data to be denoised, established different threshold equations that not only ensure better denoising effects but also overcome the deficiencies of traditional soft and hard threshold functions. However, there is a general problem in their research, i.e., the removal of noise interference will be proportionally accompanied by the elimination of real data information, resulting in data distortion after reconstruction.

In this paper, we design and achieve a new type of on-machine measuring system for the contour parameters of the drill pipe thread, so as to solve difficulties in the drill pipe thread testing. This system uses a laser displacement sensor to acquire the contour data of thread axial section. To correct the measurement error caused by the measuring point inclination angle via the laser triangulation, an inclination error model is built to compensate data in real time. To better suppress the random error interference and ensure the real contour information, a new wavelet threshold function is proposed to process data through the wavelet threshold denoising. Discrete data after denoising is segmented according to the geometrical characteristics of the drill pipe thread contour, and the regression model of each contour data is fitted by using the method of weighted total least squares (WTLS). Eventually, the thread parameters are calculated in real time to judge the thread processing quality. The experiment shows that the proposed measuring system can complete the on-machine precision testing of the drill pipe thread and improve the measurement precision and efficiency. The remainder of this paper is organized as follows. In Section 2, the scheme of the on-machine measuring system is described. In Section 3, the methods of data processing are proposed. An experiment is carried out to check the correctness of the proposed inclination error model, and the excellent performance of the improved threshold function is verified by simulation. In Section 4, the experiment results of NC50 drill pipe thread measurement are analyzed. Moreover, a contrast test on measurement accuracy is executed to validate the effectiveness of the proposed measuring system. In Section 5, the discussion of the aforementioned experiments results is given. Finally, the contributions of the proposed on-machine measuring system and data processing methods are summarized in Section 5.

## 2. The Measuring Principle and System Configuration

**Figure 1:**
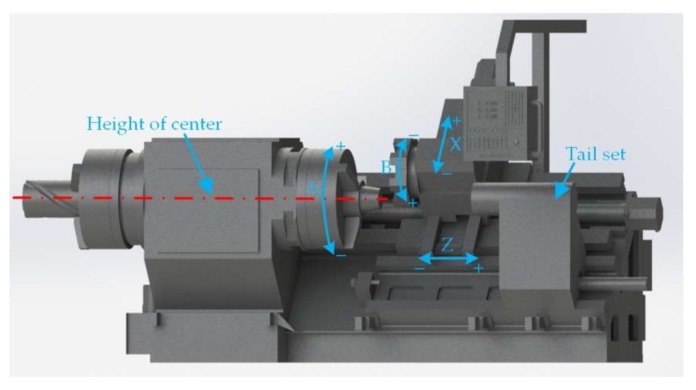
Motions of a typical four-axis machine tool.

In this paper, we propose an on-machine measuring system that takes the SCK230 drill pipe thread NC repairing machine tool, a machine tool using the Mitsubishi M70 numerical control system manufactured by Shenyang University of Technology, China, as its carrier. The basic kinematic relationship of the SCK230 CNC drill pipe thread repairing machine tool is shown in Figure 1, in which there are 4 numerical control axes like X-axis, Z-axis, A-axis, and B-axis. X-axis is the radial feeding axis of tool rest, Z-axis is the axial feeding axis of tool rest, A-axis is the rotating axis of workpiece, and B-axis is the rotating axis of tool rest. To ensure the optimum measurement performance of the laser displacement sensor, namely, that the distance from the radial measurement starting point to the thread surface to be measured is the middle of X-axis range of the sensor, the center high line of the machine tool spindle is taken as the zero position line of the sensor. When the drill pipe threads with different diameters are measured, the X-axis feeding amount in the numerical control program needs to be modified to adjust the radial measurement starting position of the sensor. In the course of numerical control repairing of the drill pipe thread, the axial extrusion length of the machined thread is controlled by the limit function of the machine tool tailstock, in which the length may be removed according actual conditions; then, as the Z-axis position of the machined thread end is known, the position of 2 mm in Z-axis positive direction of such end is selected to be the axial measurement starting point of the sensor, so as to reduce the unnecessary auxiliary time caused by the sensor due to empty travel.

**Figure 2:**
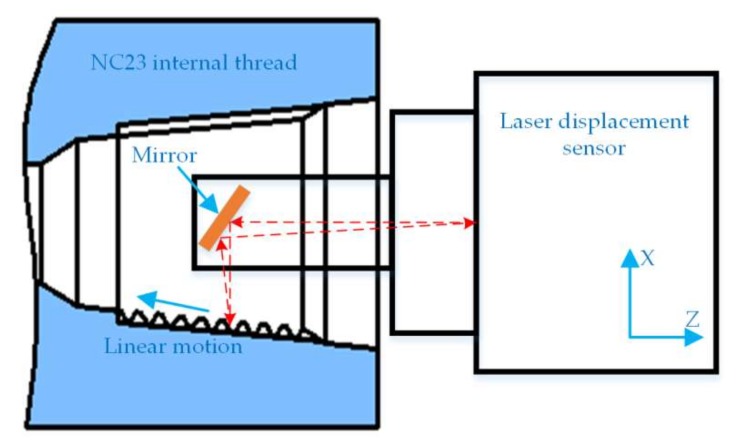
The principle of laser displacement sensor scanning.

The research on various non-contact testing devices for thread parameters shows that all the testing devices take the axial section of thread as a testing object, and then calculate the mathematical relationship between the parameters of the thread to be measured and the tested contour. Therefore, it is the core task of this system to obtain certain axial section contours of the drill pipe thread. Figure 2 presents the principle of laser displacement sensor scanning. As the minimum internal thread of the measured drill pipe is NC23, the maximum diameter only 66.97 mm, and the sensor shell 100 mm in length, it is impossible to conduct internal measurement. As a result, the mirror board is used to assist the measurement. In the measurement process, the laser displacement sensor is scanned according to the thread taper degree; the active light source is reflected vertically by the mirror board to the thread; the laser beam is reflected back to the mirror board again through the diffuse reflection of the surface to be measured; the CCD (Charge Coupled Device) in the sensor receives the diffuse reflected light signal, which is refracted back by the mirror board; and, eventually, the thread axial section contour is obtained.

**Figure 3:**
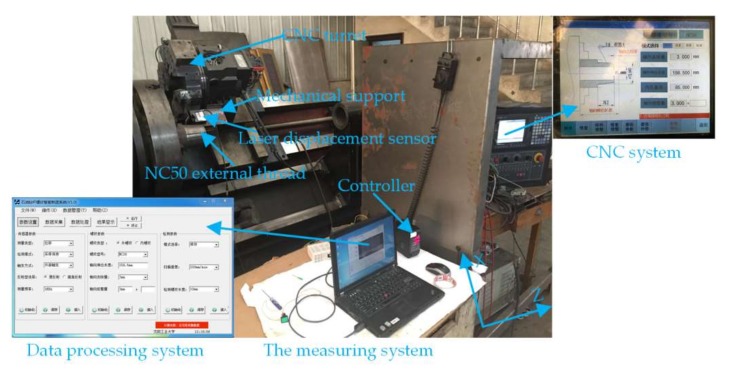
The on-machine measurement system.

The actual structure of on-machine testing system is shown in Figure 3, which mainly includes the laser displacement sensor, controller, numerical control system, and data processing system. Among that, the data processing system is developed by Shenyang University of Technology, China, and named as the first version of the drill pipe thread data processing system. As the core of the whole system, the said system has the functions of testing parameters settings, data collection, error compensation, data reconstruction, and thread parameter calculation. Further, the system is installed in a computer equipped with Intel Core I7 processor, 3.8 GHz operation speed, and 8 GB memory, and the laser displacement sensor is manufactured by Keyence, Osaka Japan with the model of LKH080, with a specific measurement range from −18 mm to18 mm, resolution 0.1 μm, and linear error ±0.02% F.S. The testing process is specified as follows: set up testing parameters in the data processing system, such as the sensor sampling frequency, the tested thread model, the tested thread length, the testing speed, etc., and synchronize the said parameters to the numerical control system through RS232 serial port. The sensor with an external signal input is installed on the CNC tool saddle by racks. When the Z-axis ball screw-driven saddle controlled by servo motor moves according to the set speed, the Z-axis ball screw encoder connects the pulse signal of certain frequencies to the input, and the sensor synchronously outputs the tested analog data to the controller through Ethernet when triggered by the valid pulse edge. Further, the data is transmitted to the data processing system after A/D conversion in the controller. After the first sensor scanning, the data processing system obtains the X and Z coordinates of thread axial section, conducts compensation and reconstruction, computes the thread parameters required according to the extracted feature points, and judges the processing quality. The results are communicated to the numerical control system via RS232 serial port, and the unqualified workpiece is corrected by lathing according to the cutter compensation, with a result that the closed-loop process of the on-machine measurement is completed.

## 3. The Data Processing Algorithm

### 3.1. The Mathematical Model and Verified Experiment of Inclination Angle Error

**Figure 4:**
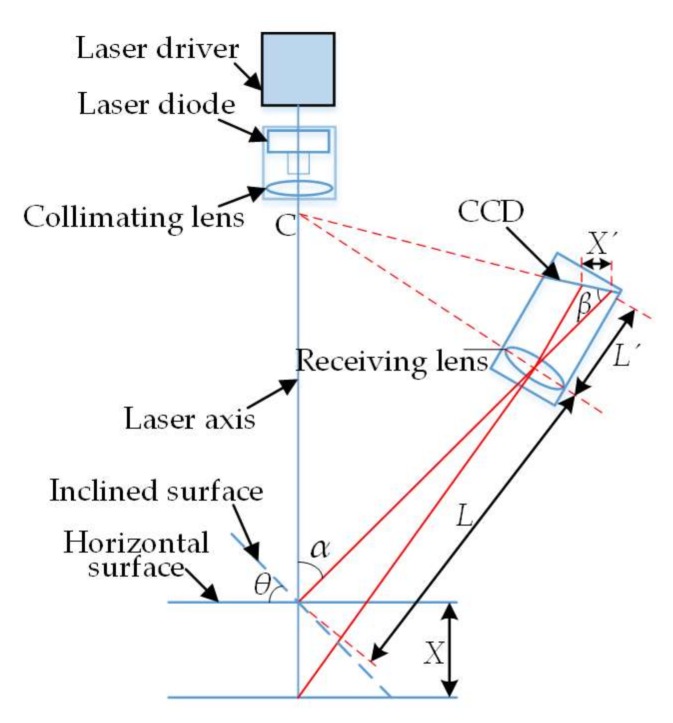
Schematic of laser triangulation.

Based on the laser triangulation principle, a laser displacement sensor is mainly composed of laser driver, laser diode, collimating lens, imaging lens, photoelectric coupling surface CCD, and signal processing circuit. The basic principle is shown in Figure 4, in which *X* is the displacement of the measured object plane, *X*’ is the displacement of the light spot image on the CCD, *α* is the angle between the optical axis of imaging lens and the laser beam, *β* is the angle between the optical axis of imaging lens and the CCD plane, *L* is the object distance of imaging lens, and *L*’ is the image distance of imaging lens. According to the theorem of similar triangles, it can be concluded that:

(1) $$X=\frac{LX^{\prime }\sin\beta }{L^{\prime }\sin\alpha -X^{\prime }\sin(\alpha +\beta )}$$

Equation (1) is the theoretical formula of laser triangulation, from which we can see the laser triangulation principle that the image displacement *X*’ presented to CCD via imaging lens reflects the actual displacement *X* of the measured object. In the actual measurement, to ensure the data acquisition accuracy of the sensor, the Scheimpflug condition must be met, i.e., the imaging lens and the CCD receiving plane intersect at the point C of the laser beam, and the Gauss’ law must be satisfied. From Figure 4 and the principle formula, the displacement measured by a sensor takes the vertical incidence of laser beam to the measured object plane as a base; nevertheless, the actual surface topography of a workpiece is so complex that the incident beam is inevitably not coincident with the surface normal. The angle between the incident beam and the surface normal is called the inclination angle *θ* (positive if clockwise), and this phenomenon is called the measuring point inclination angle. The reason for inclination error is that as the measuring point inclination angle changes the distribution of laser scattering field, the light received by the imaging lens is changed accordingly, with the result that the light spot centroid imaged in the CCD is not coincident with that at the time of vertical incidence. However, the sensor still calculates the displacement, providing there is no measuring point, and eventually the error is caused. Therefore, an inclination error model is to be built to effectively improve the data acquisition accuracy of the laser displacement sensor.

**Figure 5:**
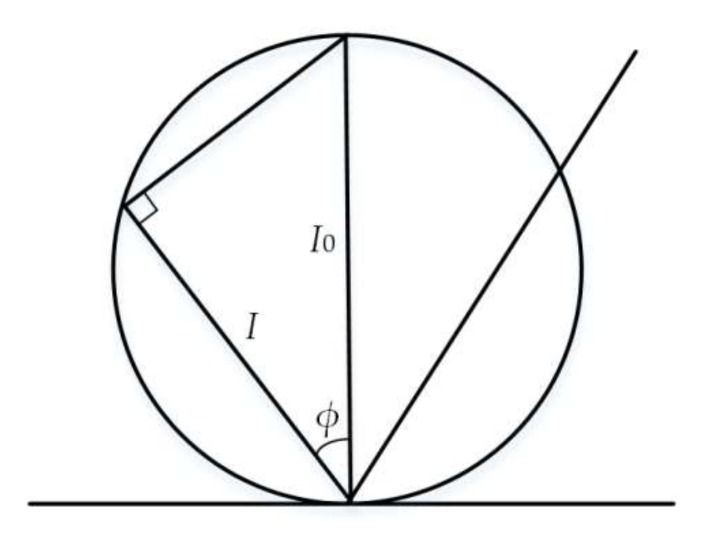
Lambert surface scattering field distribution.

Under ideal conditions, the measured object plane is considered as a diffuse surface without absorption. According to the Beer-Lambert Law (as shown in Figure 5), the spatial distribution of light scattering field is

(2) $$I=I_{0}\cos\phi$$

in which *φ* is the angle between the scattering beam and the normal of the object plane; *I* is the power of the scattered light, which is in the angular direction with the normal of the object plane; and *I*_0_ is the ideal power of the scattered light in the normal direction.

**Figure 6:**
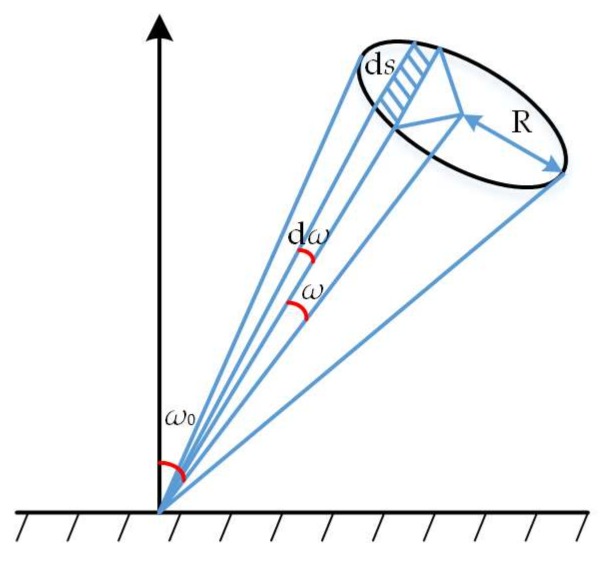
Schematic of a beam for received light.

As shown in Figure 6, we set d*s* as the imaging lens surface vertical to the scattered light receiving surface, and then the light received in the unit time is

(3) $$dE=I\cos(\omega_{0}-\omega )d\sigma$$

in which d*σ* is the solid angle of the surface to the incidence point, *ω*_0_ is the angle between the imaging lens axis and the normal of the measured object, and *ω* is the angle between the imaging lens axis and the surface boundary. According to the geometrical relationship of the receiving surface in Figure 6 and in combination with the Beer-Lambert Law, the light received by the imaging lens is

(4) $$E=\pi I_{0}\frac{R^{2}}{L^{2}}\left(1+2\frac{X}{L}\cos\omega_{0}\right)\cos(\omega_{0}-\omega )$$

in which *R* is the radius of imaging lens. Further, as *ω* is very small, we approximately deem that sin*ω* = tan*ω* and *ω*_0_ = *α* − *θ*, and hence it can be deduced that the angular position *ω*_1_ of the light centroid line in imaging lens is

(5) $$\omega_{1}=2\frac{XR^{2}}{L^{3}}\cos\alpha \cdot \tan(\alpha -\theta )$$

The projection point of the centroid line on the CCD is the centroid position of converged light spots, and the method for determining the position of the light centroid line on the CCD is given below.

**Figure 7:**
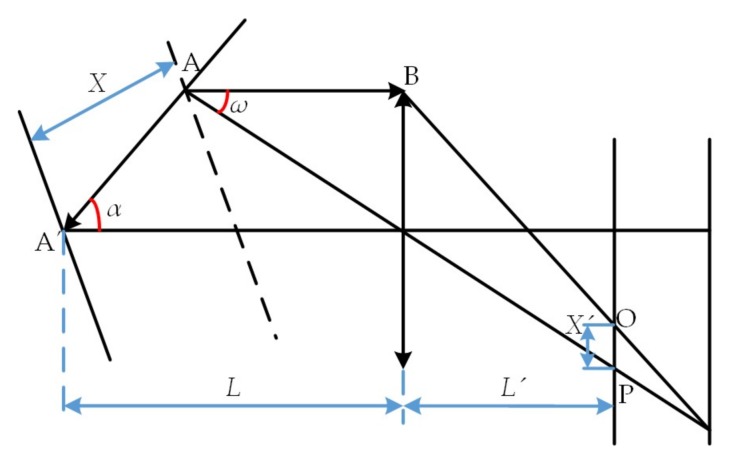
Schematic of light spot centroid.

In the said paragraph, it is stated that in case of a measuring point inclination angle, the light centroid on the CCD relatively deviates from the geometric center of converged light spots, and the amount of deviation is the measuring point inclination error. Figure 7 gives a schematic diagram of the light spot centroid, in which the point P on the CCD is the centroid position of the centroid line AB through the refraction of imaging lens. According to the geometrical relationship shown in Figure 7, it can be reduced that the distance between the imaging point P of the light centroid line on the CCD and the imaging lens axis is

(6) $$OP=\frac{L^{\prime }X\sin\alpha }{L-X\cos\alpha }-\frac{L^{\prime }X\sin\alpha }{L}\omega^{\prime }$$

Combined with Equations (5) and (6), it can be concluded that when the incidence is vertical, i.e., the measuring point inclination angle is 0, *ω*´ = *ω*_1,_ and *θ* = 0, and when the inclination is accompanied by an inclination angle, i.e., the measuring point inclination angle is *θ*, *ω*´ = *ω*_1_ and *θ* ≠ 0, and the inclination error is

(7) $$\begin{array}{l} \varepsilon_{\theta }={OP|}_{\omega^{\prime }=\omega_{1},\theta \neq 0}-{OP|}_{\omega^{\prime }=\omega_{1,\theta =0}}=2\frac{X^{2}R^{2}}{L^{3}}\cos\alpha \left[\tan\alpha -\tan\left(\alpha -\theta \right)\right] \end{array}$$

From Equation (7), it can be concluded that the measuring point inclination error is only related to the two variables of object plane displacement *X* and measuring point inclination angle *θ*, and the rest are the optical structural parameters of the laser displacement sensor. Through the analysis, it can be concluded that when the measuring point inclination angle is fixed, the measurement error increases with the increase of the measuring distance; when the measuring distance is fixed, the measurement error increases with the increase of the measuring point inclination angle; when *θ* > 0, the measurement error varies with the object plane displacement in the same direction; but when *θ* < 0, the measurement error varies with the object plane displacement in the opposite direction.

The said results are a quantitative model for the inclination error of the laser displacement sensor. Although some assumptions are made in the derivation process, and some parameters are approximated, the theoretical results are still somewhat deviated from the actual measurement values. However, in the engineering application, it is true that the quantitative model can effectively facilitate the data acquisition accuracy of the laser displacement sensor.

The inclination error experiment is composed of a four-axis vertical machining center, a laser interferometer, a laser displacement sensor, a sine bar, and a computer. The laser interferometer is manufactured by Renishaw, London England with the model of XL-30, with the specific measuring range of 30 m, the resolution of 0.01 μm, and the measurement error of ±0.1 ppm. The parameters of the laser displacement sensor and computer are both given in the said paragraphs.

**Figure 8:**
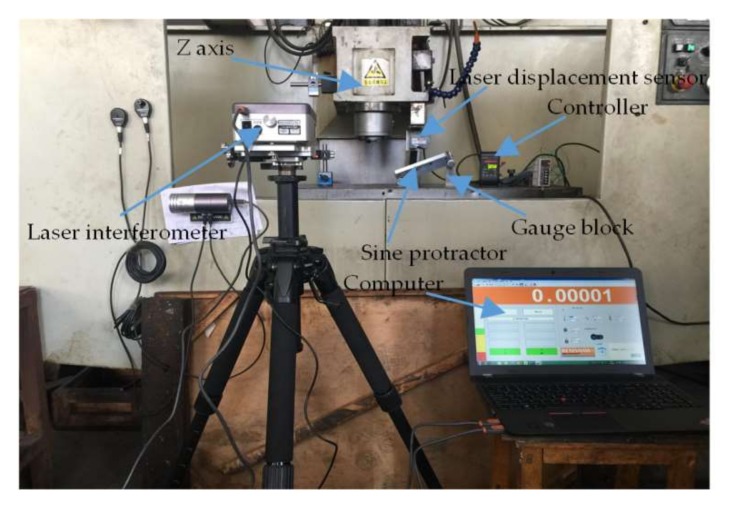
The experiment of inclination error.

**Figure 9:**
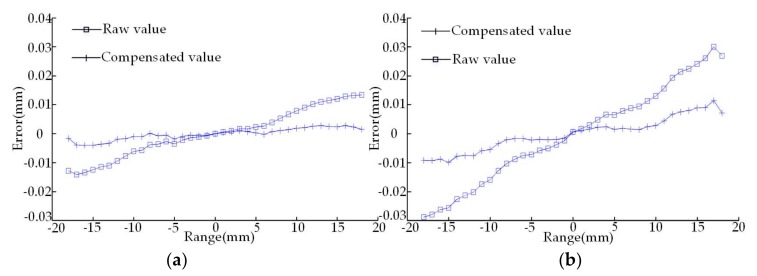
Inclination angle errors and compensation graph. (**a**) The experiment of 20° inclination angle error; (**b**) the experiment of 60° inclination angle error.

As the experimental process is shown in Figure 8, the optical path components of the sensor and the interferometer are installed on the Z-axis and work platform of the machining center; the sine bar is placed on the work platform right below the sensor, and it can be jacked a certain angle by a gauge block; the sensor readings are shown on the controller; and the interferometer readings are displayed on the computer. After the start of the experiment, when the sensor and the interferometer move each 1 mm within the range of the laser displacement sensor, their values are recorded respectively. As the half of thread angle of the drill pipe is 30°, the measuring point inclination angle in the flank is 60°. For purpose of the preparation of the subsequent thread measurement experiment on the basis of verifying the inclination error model, the sine bars with the inclination angles 20° and 60° are built, along with the inclination error compensation shown in Figure 9a,b.

Figure 9 demonstrates that when the inclination angle of the object plane is fixed, the measurement error of the laser displacement sensor increases with the increase of the measuring distance; and when the measuring distance is fixed, the measurement error of the laser displacement sensor increases with the increase of the inclination angle of the object plane. The inclination error experiment proves that the proposed mathematical model of inclination error is both accurate and effective. Through this model, we can not only quantitatively calculate the measurement error of the laser displacement sensor caused by the inclination angle but also significantly improve the data acquisition accuracy through error compensation.

### 3.2. An Improved Wavelet Threshold Denoising Algorithm and Simulation

The point cloud data acquired by the laser displacement sensor is affected by the surface properties and geometrical topography of thread and the structure of the measuring system, resulting in random noises in the measured data. Therefore, it is required to denoise the original data. In this section, we process the noises in the thread contour data by using the wavelet denoising method applied in the signal analysis. In the wavelet threshold denoising, the quantified threshold function is the key to denoising effects. At present, the threshold functions are mainly:

(1) The hard threshold function:
  (8) $$\eta \left(x,\lambda \right)=\{\begin{array}{c} x\\ 0 \end{array}\begin{array}{cc} , & \left|x\right|\geq \lambda \\ , & \left|x\right|<\lambda \end{array}$$
(2) The soft threshold function:
  (9) $$\eta \left(x,\lambda \right)=\{\begin{array}{ll} x-\lambda , & x\geq \lambda \\ 0, & \left|x\right|<\lambda \\ x+\lambda , & x\leq -\lambda \end{array}$$
(3) The improved threshold function in Reference [26]:
  (10) $$\eta \left(x,\lambda \right)=\{\begin{array}{ll} \mathrm{sgn}\left(x\right)\left\{\left|x\right|-\frac{\lambda }{\exp^{3}\left[m\left(\left|x\right|-\lambda \right)/\lambda \right]}\right\}, & \left|x\right|\geq \lambda \\ 0, & \left|x\right|<\lambda \end{array}$$

in which *x* is wavelet coefficient, $\lambda =\sqrt{2\log\left(N\right)}$ is threshold, *σ* is the noise standard deviation in the point cloud data (i.e., noise intensity), and *N* is data amount. When it comes to the solution of actual problems, we need to estimate the unknown parameters.

The said three threshold functions all have some drawbacks, although they achieve certain denoising effects. In Equation (8), the hard threshold function is discontinuous in x=±λ, which leads to the reconstruction oscillation of the wavelet coefficients with poor denoising degrees; in Equation (9), the real wavelet coefficients of the soft threshold function deviate from the estimated wavelet coefficients, which results in the poor approximation of the reconstructed data to the real data; and Equation (10) presents a multitude of improved threshold functions that improve the phenomenon of discontinuity but still fail to solve the deviation of wavelet coefficients in the wavelet decomposition. Therefore, there still remains data distortion to various degrees after denoising by these methods.

In view of the deficiencies of the said wavelet threshold denoising algorithm, a new wavelet threshold function is proposed, which cannot only control the constant deviation of wavelet coefficients but also fully ensure the function is continuous and high-order derivative within the threshold range. The new threshold function is

(11) $$\eta \left(x,\lambda \right)=\{\begin{array}{ll} nx-0.4\frac{m{\left(\mathrm{sgn}\left(x\right)\lambda \right)}^{a}}{{\left(nx\right)}^{a-1}}+m\mathrm{sgn}\left(x\right)\lambda , & \left|x\right|>\lambda \\ 0.4m\frac{{\left(n\left|x\right|\right)}^{b}}{\lambda^{b-1}}\mathrm{sgn}\left(x\right), & \left|x\right|\leq \lambda \end{array}$$

in which coefficients *a*, *b* (*a*, *b* > 0) are the adjusting parameters of the new threshold function, and when the random error of point cloud data is removed, the adjustment of the said two coefficients can change the variation mode of threshold function. The adjustment of coefficients *m* (0 ≤ *m* ≤ 1), *n* (0 ≤ *n* ≤ 1), which are approximated parameters of the reconstructed data in interval range, not only ensure the global continuity of the hard threshold function without oscillation but also effectively control the constant deviation of wavelet coefficients of the soft threshold function in the wavelet decomposition. Finally, the function still has all the advantages of traditional threshold functions. According to the said analysis, the new wavelet threshold function not only ensures the denoising effects but also avoids killing the real data. Moreover, the new wavelet threshold function is more flexible and performs better.

**Figure 10:**
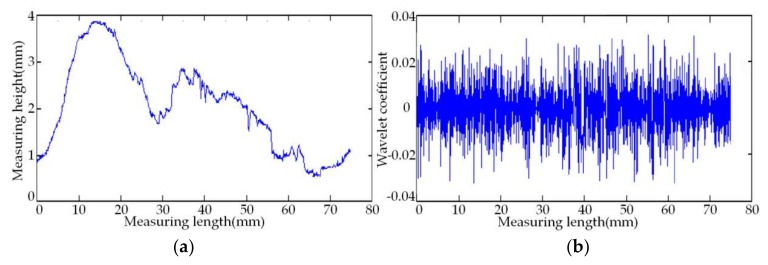
The original data and its wavelet coefficient. (**a**) The original data of denoising algorithm; (**b**) the wavelet coefficient of original data.

As shown in Figure 10, the simulation experiment employs a set of measuring point cloud data with random errors, as well as the original data and its wavelet-decomposed coefficients. Sym4, sym6, and sym8 in Symlets wavelets and db6, db8, and db10 in Daubechies wavelets are selected as the mother wavelet functions that are used to denoise the point cloud data. After comparison of the results, the sym8 wavelet is finally chosen to make the five-level wavelet decomposition of the set of data.

**Figure 11:**
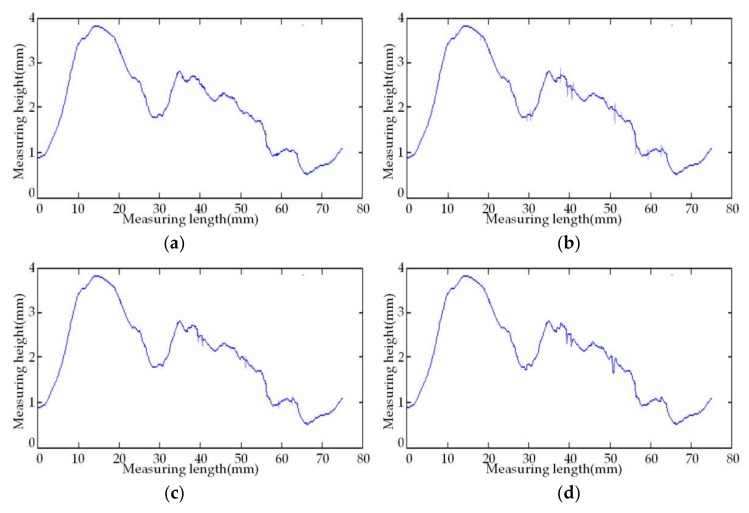
The effect comparison of different threshold functions in data denoising. (**a**) The soft threshold function; (**b**) the hard threshold function; (**c**) the improved threshold function in reference [26]; (**d**) the new threshold function in this paper.

In order to prove the new threshold function’s ability to denoise the random errors in the noisy cloud data, we respectively employ the traditional soft and hard threshold functions, the improved threshold function in Reference [26], and the proposed self-adaptive wavelet threshold function to denoise the data in Figure 10. In this paper, the proposed wavelet threshold denoising algorithm uses the heuristic threshold rule (the Heursure rule) to carry out the self-adaptive threshold denoising. The parameters of the proposed improved threshold function are set to be *a* = 5, *b* = 2, *m* = 0.6, *n* = 0.9, and the experimental data after denoising are shown in Figure 11.

Compared with the said four graphs, it can be clearly found that the soft threshold function performs better in denoising; nonetheless, it kills the real data details in a more severe way; the hard threshold function performs worse in denoising, and it cannot well filter random errors; and the improved threshold function in Reference [26] solves the oscillation phenomenon caused by the discontinuity of the hard threshold function in x=λ, improving the level of data denoising and various performance indicators. However, some real data details are filtered while enhancing the denoising capacity. The new self-adaptive wavelet threshold function proposed in this paper successfully solves the said problems. It not only avoids the oscillation phenomenon caused by the discontinuity but also controls the false deletion of the high-frequency signal in the real data information, improving the data reconstruction accuracy. Additionally, it achieves significant effects in the denoising and the maintenance of data reality and integrity.

In order to accurately compare the denoising effects of the traditional soft and hard threshold functions with the said two threshold functions, it is necessary to introduce a unified and objective assessment criterion. In this paper, quantitative analysis is conducted in accordance with three assessment indexes for the denoising performance, namely, signal-to-noise ratio, root-mean-square error, and smoothness:

(1) SNR (Signal-to-noise ratio) is defined as follows:
  (12) $$\mathrm{SNR}=10\mathrm{lg}\left[\frac{\sum_{i=1}^{n}x^{2}\left(t\right)}{\sum_{i=1}^{n}{\left(x\left(t\right)-\hat{x}\left(i\right)\right)}^{2}}\right]$$
(2) RMSE (Root-mean-square error) of original data and denoised data is defined as follows:
  (13) $$\mathrm{RMSE}=\sqrt{\frac{1}{n}\sum_{i=1}^{n}{\left(x\left(i\right)-\hat{x}\left(i\right)\right)}^{2}}$$
(3) Smoothness is defined as follows:
  (14) $$R=\frac{\sqrt{\sum_{n-1}{\left[\hat{x}\left(i+1\right)-\hat{x}\left(i\right)\right]}^{2}}}{\sqrt{\sum_{n-1}{\left[x\left(i+1\right)-x\left(i\right)\right]}^{2}}}$$

in which x(i) is original data, $\hat{x}\left(i\right)$ is the data after the wavelet function denoising, and *n* is the number of data points. The definition is shown as follows: the greater the SNR (unit: dB) of the data after denoising is, the smaller the RMSE and the smoothness *R* value are. This indicates that the data after denoising is much closer to the original data, with better denoising effects.

The values of the said three assessment indexes after denoising by four threshold functions are shown in Table 1. It can be seen from the table that the new self-adaptive wavelet threshold function proposed in this paper significantly outperforms other threshold functions in denoising the point cloud data.

### 3.3. Thread Contour Partitioning, Fitting, and Parameters Calculation

**Figure 12:**
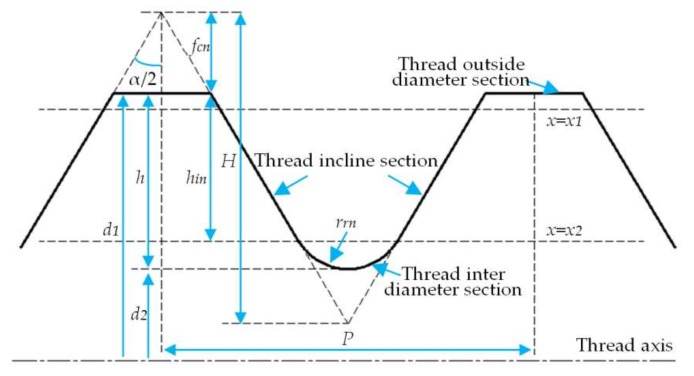
The contour curve of drill pipe thread.

As shown in Figure 12, the contour of the drill pipe thread is composed of threaded outside diameter and a rounded root and flank, and it has a periodic complex surface. Therefore, large deviations will be caused if the contour data after filtering is directly used to calculate the fitted curve. Accordingly, the discrete data points are reasonably segmented according to the geometrical characteristics of the contour, and then the regression models fitted in each section are combined to obtain the thread parameters.

After the comparison of all the measuring points after denoising, the maximum coordinate value *x*_max_ along the vertical axis can be obtained, and the corresponding measuring point should be located on the outside diameter of the thread contour. *x*_1_ = *x*_max_ − *f* (*f* is the length of the tolerance band of thread outside diameter) is taken as the first horizontal dividing line, which divides the data points into two parts, and the point in the upper part of the dividing line *x* = *x*_1_ is taken as the point domain *P*_1_ of the thread outside diameter. By virtue of the standard parameters of the drill pipe thread given in Reference [27], the height *h*_in_ of the flank can be calculated.

(15) hin=H−fcn+rrn(sinα2−1sinα2)

in which *H* is the original triangle height of thread profile, *f*_cn_ is the depth of truncation of crest, *r*_rn_ is the radius of rounded root, and *α*/2 is the half of thread angle. After taking the second dividing line *x*_2_ = *x*_max_ − *h*_in_ and dividing the remaining data points, we can get *r* flank domains *P*_2*i*_ (*i* = 1, 2,…, *r*) at the upper part of the dividing line *x* = *x*_2_ and s rounded root domains *P*_3j_ (*j* = 1, 2,…,*s*) at the lower part of the dividing line *x* = *x*_2_.

Actually, the weighted least squares (WLS) method is the most commonly-used data fitting method in engineering; this algorithm only considers the data vector and ignores the possible errors in the coefficient matrix. Therefore, how to deal with the error of the coefficient matrix in the fitting process is of great significance. Therefore, in this paper, the WTLS in Reference [28] is employed to calculate the regression model of the contour data in each section.

First, the regression models of data segments of three different contours are given, and the regression coefficients of each fitting segment are calculated according to the WTLS in Reference [28]. The crest, flank, and rounded root are shown in the following equation:

(16) $$\{\begin{array}{c} X_{1}\left(z\right)=a_{1}+b_{1}z\begin{array}{cc} , & \left(z,x\right)\in P_{1} \end{array}\\ X_{2i}\left(z\right)=a_{2i}+b_{2i}z\begin{array}{cc} , & \left(z,x\right)\in P_{2} \end{array}\\ X_{3j}\left(z\right)=a_{3j}+\sin\left(b_{3j}z+c_{3j}\right)\begin{array}{cc} , & \left(z,x\right) \end{array}\in P_{3j} \end{array}$$

**Figure 13:**
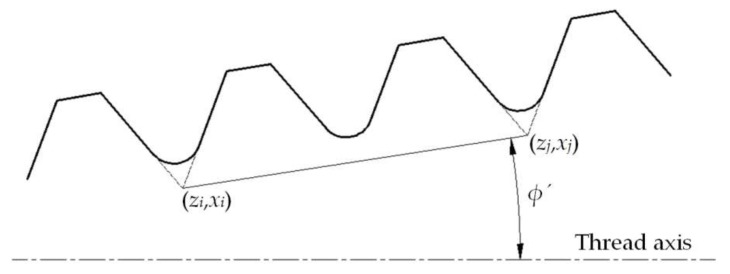
Diagram of taper calculating.

When the contour after segmentation is fitted, the regression model of each data segment is used to calculate the target parameters required by the system, namely, pitch *P*, half of thread angle *α*/2, thread height *h,* as shown in Figure 13.

The pitch can be obtained at the intersection point of the thread outside diameter and the flank fitted curve:

(17) $$P_{i}=\cos\phi \left(\frac{a_{1}-a_{2,2i-1}}{b_{2,2i+1}-b_{1}}-\frac{a_{1}-a_{2,2i-1}}{b_{2,2i-1}-b_{1}}\right)\begin{array}{cc} , & \left(i=1,2,\cdots ,\frac{1}{2}r-1\right) \end{array}$$

in which *φ* is the half of standard cone angle.

The half of thread angle can be calculated by the slope of the flank curve:

(18) $$\frac{\alpha_{i}}{2}=\left|\arctan b_{2,i}\right|\begin{array}{cc} , & \left(i=1,3,\cdots ,r-1\right) \end{array}$$

The tooth height is defined as the distance between the thread outside diameter and the bottom of the rounded root (*h* = *d*_1_ − *d*_2_), and its expression is

(19) $$h_{j}=\frac{b_{2,i}a_{1}-b_{1}a_{2,i}}{b_{2,i}-b_{1}}-a_{3,j}+1\begin{array}{cc} , & \left(i=1,3,\cdots ,r-1\;j=1,2,\cdots ,s\right) \end{array}$$

As shown in Figure 13, taper can be obtained through the calculation of the half of cone angle *φ*′:

(20) $$T=2\tan\phi^{\prime }=2\frac{\sin\phi \left(z_{i}-z_{j}\right)+\cos\phi \left(x_{i}-x_{j}\right)}{\cos\phi \left(z_{i}-z_{j}\right)-\sin\phi \left(x_{i}-x_{j}\right)}\begin{array}{cc} , & \left(i\neq j\right) \end{array}$$

in which $\left(z_{i},x_{i}\right)$, $\left(z_{j},x_{j}\right)$ are the intersection coordinates of the flank curve.

## 4. Results

**Figure 14:**
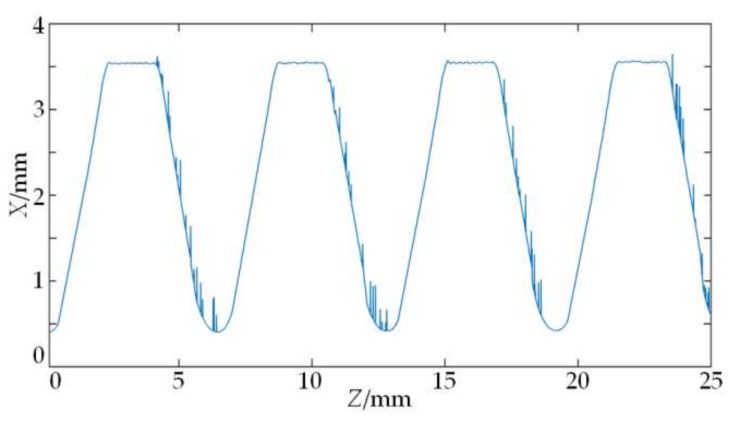
Original contour data of NC50 thread.

**Figure 15:**
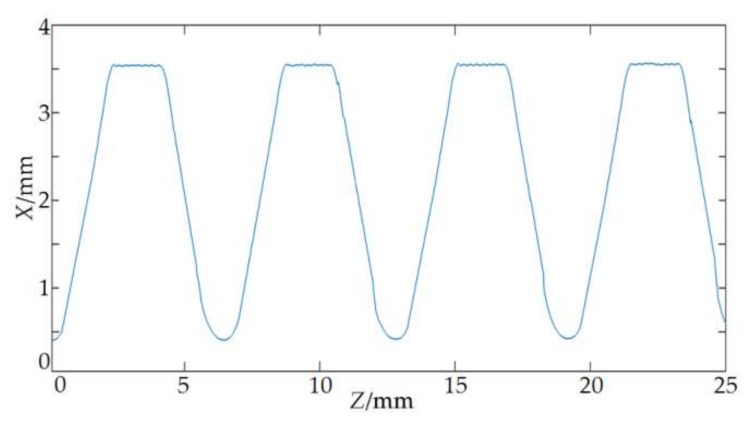
Contour data after compensation and denoising.

The on-machine measuring system for the parameters of the drill pipe thread designed in this paper is used to measure the parameters of the NC50 drill pipe thread lathed in SCK230 drill pipe thread repairing machine tool, and the scanned original data is shown in Figure 14. The contour of the thread axial section is expressed by the 2D discrete curve $P=\left[z_{i},x_{i}\right],i=1,2,\cdots ,3731$. Since the thread contour is a large inclined periodic surface, the original data of the flank should compensate for the inclination error. First, according to the degree of half of thread angle of the drill pipe, the inclination angles of two thread flanks relative to the horizontal plane can be known. Then, the theoretical distance between each sampling point on the thread surface and the laser displacement sensor is calculated from the number *i* of each data point, the interval of the trigger pulse, and the relative position of the laser displacement sensor to the thread surface. Finally, the said inclination degree and theoretical distance are put into Equation (7) to calculate the amount of inclination error compensation for each data point in the flank section. After the inclination error compensation is made for the curve, the wavelet denoising based on the improved threshold function is employed. The contour of NC50 thread axial section after denoising is shown in Figure 15, and it proves that the noises are obviously weakened after smoothness.

According to the said thread contour data segmentation method, 3150 discrete domains are segmented by filtering, which are divided into 1 crest domain *P*_1_ = {[2.340, 4.133], [8.720, 10.447], [15.067, 16.847], [21.473, 23.213]}, 8 flank domains, i.e., *P*_21_ = [0.372, 2.340], *P*_22_ = [4.133, 6.102], *P*_23_ = [6.752, 8.720], *P*_24_ = [10.447, 12.416], *P*_25_ = [13.099, 15.067], *P*_26_ = [16.847, 18.816], *P*_27_ = [19.505, 21.473], *P*_28_ = [23.213, 25.182], and 3 rounded root domains *P*_31_ = [6.102, 6.752], *P*_32_ = [12.416, 13.099], *P*_33_ = [18.816, 19.505], and the regression model of the curve in each section is fitted according to the WTLS method, with the fitted coefficients shown in Table 2.

The residual sum of squares (RSS) $r_{k}=\sum_{k=1}^{12}e_{k}^{2}$ is used to measure the regression degree of the fitted curve. According to the RSS of the regression model in each section in Table 2, it can be seen that the fitted contour regression in the positions of crest and root is better, but the regression of the fitted curve is worse at two sides of flank. This is because the angle between the incident light and the reflected light by the laser triangulation method is much closer to half of thread angle. In addition, the regression on the left of flank obviously outperforms that on the right due to different reflection directions of the laser beam.

**Figure 16:**
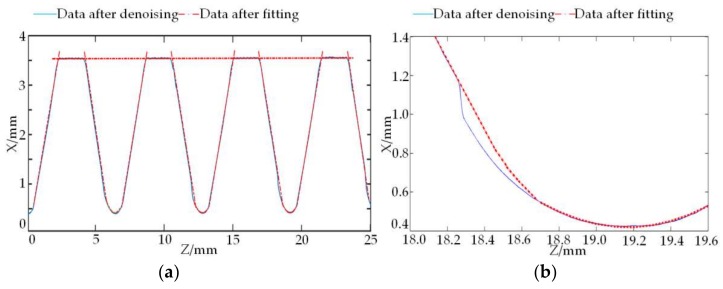
Contour fitting and partial enlarged drawing. (**a**) The contour data after fitting; (**b**) partial enlarged drawing of thread inter diameter section.

Figure 16 is the fitted curve and partial enlarged drawing of the contour of NC50 thread axial section, in which Figure 16b is the partial enlarged drawing of Figure 16a at the intersection of the flank and the rounded root. It can be seen from Figure 16a that the scanned contour and the fitted contour are fitted closely together, and the regression of the fitted curve in each section performs well, which proves that the filtering algorithm effectively eliminates interference noises. Further, it can be seen from Figure 16b that the fitted curve also solves the problem that the light spot image cannot be received by the optical coupler of the sensor due to the reflection of the incident light that the dead zone caused, resulting in the measured data deviation.

**Figure 17:**
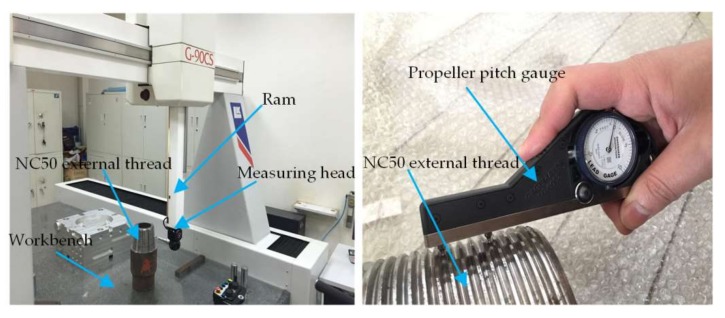
Measuring experiment for comparison.

In order to test the capability of the on-machine measuring system, the LK-G90CS three-coordinate measuring machine (CMM) which is manufactured by LK, Derbyshire England, the Gagemaker individual thread parameter gauge (ITPG), and the on-machine measuring system (OMS) are used respectively to measure the 1st to 4th thread pitch *P*_1_, *P*_2_, *P*_3_; height *h*_1_, *h*_2_, *h*_3_; half of thread angle *α*_1_/2, *α*_2_/2, *α*_3_/2, *α*_4_/2; and taper *T* of the NC50 drill pipe thread. Among these, the OMS method is divided into two types: OMS 1 and OMS 2. Additionally, OMS 1 is used to calculate the sample data by using the inclination error compensation algorithm and the wavelet threshold denoising algorithm proposed in this paper, while OMS 2 is used to directly calculate the sample data without using the said algorithms. Figure 17 shows a measuring experiment for comparison. After 30 times of measurement by the said measuring methods, the mean of the 1st to 4th thread parameters and relevant nominal values and tolerance bands are shown in Table 3. Strictly speaking, the measurement accuracy of the three-coordinate measuring machine is so high that the measured data can be served as the real parameters of the actual machined thread. In Table 3, error 1 is the difference between the testing results of the proposed on-machine system and data processing method and those of the CMM method, i.e., the testing accuracy of the OMS 1 method; error 2 is the difference between the testing results of the only on-machine testing system and those of the CMM method, i.e., the testing accuracy of the OMS 2 method; and error 3 is the difference between the testing results of the ITPG method mentioned in Introduction, which is widely used in current oilfields and those of the CMM method, i.e., the testing accuracy of the ITPG method; and nominal values are the standard values of each thread parameter, and its tolerance zones are used to judge whether a machined thread is qualified.

The repeatability of measurement results in the industrial measuring equipment is extraordinarily important, and the repeatability of the measuring equipment refers to, under the same measurement condition, the consistency of measuring results obtained after multiple measurements of the same object. In the error theory, the size of the experimental standard difference S(*x*) calculated by the repeatability limit and the Bessel Equation (21) is used to quantitatively describe the repeatability of the measuring equipment. The greater the S(*x*) is, the greater the repeatability limit is; the more scattered the measurement results are, the worse the consistency is and the lower the repeatability is. In contrast, the smaller the repeatability limit is, the more concentrated the measurement results are, the better the consistency is, and the higher the repeatability is.

(21) $$S\left(x\right)=\sqrt{\frac{1}{n-1}\sum_{i=1}^{n}{\left(x_{i}-\bar{x}\right)}^{2}}$$

in which *x_i_* is the result obtained for the *i* time and $\bar{x}$ is the arithmetic mean for *n* times of measurement.

**Figure 18:**
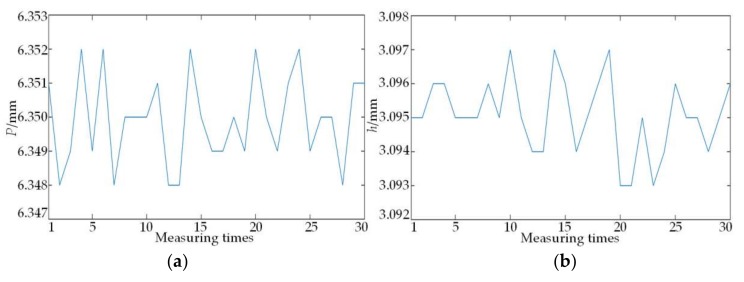
Repeated measuring results. (**a**) Repeated measuring results of pitch; (**b**) repeated measuring results of height.

Under the same condition, the on-machine measuring system is used to continuously measure the 2nd to 3rd thread pitch *P* and the 2nd thread height *h* of the standard NC50 thread ring gauge 30 times, and the results are shown in Figure 18.

## 5. Discussion

The mean of each parameter obtained by the system after 30 times of measurement is compared with that of other measuring methods. After analysis of the data in Table 3, it can be concluded from the comparison of the testing accuracy of the OMS 1 and OMS 2 methods (comparison of results of error 1 and error 2) that the data processing algorithm proposed in this paper has significantly improved the testing accuracy of the on-machine testing system for the contour parameters of the drill pipe thread. After comparison with nominal values, it can be concluded that the differences are all within the tolerance range stated in the national standard, which indicates that the measured NC50 drill pipe thread is well repaired. Compared with the experimental data obtained by the individual thread parameter gauge (comparison of results of error 1 and error 3), the measurement accuracy of the on-machine measuring system is markedly higher than that of the traditional individual thread parameter gauge, and it also solves the problem that the half of thread angle cannot be tested. Compared with the measuring results obtained by the three-coordinate measuring machine, it can be concluded that the system measurement accuracy can reach ±6 μm, and as the sensor feeding speed during the measurement is F = 200 mm/min and the length of the tested thread is 25 mm, then each testing costs 7.8 s. In summary, among the three testing methods, the CMM method is of the highest testing accuracy, followed by the OMS method. Additionally, the ITPG method performs the worst and, compared with the fast, automatic, and on-machine testing method proposed in this paper, both of the CMM and ITPG methods require a large amount of manual work, resulting in much poorer performance in testing efficiency than the OMS method. For instance, if we adopt the CMM method, we should cut the thread from the drill pipe to carry out the off-machine measurement. It is obvious that the CMM method requires a large amount of testing time so that it is only suitable for the laboratory sampling. Although the ITPG method can perform the on-machine measurement of drill pipe thread parameters, the testing efficiency will be inevitably affected on account of contact-type manual measurement. Therefore, although the proposed OMS method performs worse than the CMM method in accuracy, the former is obviously more suitable for measuring contour parameters of the drill pipe thread, and it greatly outperforms the widely used ITPG method in terms of measuring way, testing efficiency, and testing accuracy. In addition, it can be found from comparison of the results of error 1 and error 2 that the data processing algorithm proposed in this paper significantly improves the testing accuracy of the on-machine testing system.

The repeatability tests show that the maximum deviation shown in the Figure 18 is 4 μm, and hence the repeatability limit of the on-machine measuring system ≤4 μm, and then according to Equation (21), S(*p*), and S(*h*) calculated based on 30 times of measurement are respectively 0.0013 and 0.0012. The same procedure is adopted to obtain the result that both the half of thread angle and taper have more than low experimental standard deviation. Accordingly, it can be concluded that the on-machine testing system has high repeatability of measurement.

## 6. Conclusions

In this paper, we propose a precise and on-machine testing method for the drill pipe thread. By using favorable numerical control resources, this method enables us to test unqualified workpieces timely and then make correction quickly. In terms of data acquisition accuracy and reconstruction accuracy of the laser displacement sensor, the measurement error is compensated effectively by virtue of the inclination error model. Further, the improved wavelet threshold function is proposed and successfully used to denoise the point cloud data of thread contour, with the result that the data quality is dramatically improved. The method proposed in this paper plays a certain guiding role, for it can also be extended to the fields of reverse engineering and precision testing. The SCK230 drill pipe thread repairing machine tool with this testing method has been put into use in Changqing, Liaohe, and other oilfields in China. After actual operation, it proves that the measurement accuracy of this method is ±8 μm, and the testing of 25 mm thread costs 7.8 s, which can meet the requirements for the precise control and quick testing of a large-sized workpiece during the on-line production.

## Figures and Tables

**Table 1:** The comparison of de-noising results for different threshold functions.

Denoising Method	SNR/db	RMSE	*R*
Hard threshold function	23.7252	4.1062	0.2333
Soft threshold function	22.3917	4.9006	0.2152
Improved threshold function	27.5626	3.8963	0.2218
This paper	29.8651	3.1062	0.1962

**Table 2:** Coefficient of regression models.

	*a*	*b*	*c*	*r*
*X*_1_	3.541	−0.001		0.007
*X*_21_	−0.440	1.735		0.132
*X*_22_	11.540	−1.745		0.226
*X*_23_	−11.565	1.743		0.066
*X*_24_	21.597	−1.714		0.256
*X*_25_	−23.076	1.772		0.072
*X*_26_	34.169	−1.762		0.106
*X*_27_	−34.451	1.775		0.083
*X*_28_	46.214	−1.826		0.233
*X*_31_	1.417	1.159	−9.011	0.008
*X*_32_	1.413	−1.087	12.330	0.002
*X*_33_	1.419	−1.091	19.333	0.002

**Table 3:** Comparison of the three measuring methods.

	*P*_1_/mm	*P*_2_/mm	*P*_3_/mm	*h*_1_/mm	*h*_2_/mm	*h*_3_/mm	*α*_1_/2(°)	*α*_2_/2(°)	*α*_3_/2(°)	*α*_4_/2(°)	*T*
CMM	6.353	6.349	6.350	3.130	3.122	3.125	29°57′	30°02′	30°17′	29°20′	0.1682
ITPG	6.344	6.357	6.359	3.120	3.127	3.121					0.1677
OMS 1	6.347	6.351	6.358	3.122	3.120	3.120	30°03′	29°58′	30°24′	29°28′	0.1680
OMS 2	6.345	6.355	6.362	3.120	3.129	3.118	30°39′	29°37′	30°47′	29°53′	0.1677
Error 1	−0.006	0.002	0.008	−0.008	−0.002	−0.005	6′	4′	7′	8′	−0.0002
Error 2	−0.008	0.006	0.012	−0.010	0.007	−0.007	42′	25′	30′	33′	−0.0005
Error 3	−0.009	0.008	0.009	−0.010	0.005	−0.004					−0.0005
Nominal value	$6.35_{-0.01}^{+0.01}$	$6.35_{-0.01}^{+0.01}$	$6.35_{-0.01}^{+0.01}$	$3.095_{0}^{+0.12}$	$3.095_{0}^{+0.12}$	$3.095_{0}^{+0.12}$	${30{}^{\circ}}_{-45}^{+45\prime }$	${30{}^{\circ}}_{-45\prime }^{+45\prime }$	${30{}^{\circ}}_{-45\prime }^{+45\prime }$	${30{}^{\circ}}_{-45\prime }^{+45\prime }$	$0.167_{0}^{+0.0025}$

## References

[1] Lin T.J., Zhang Q., Lian Z.H., Liu Y.G., Zhang Y., Chen Y. (2016). Multi-axial fatigue life prediction of drill collar thread in gas drilling. Eng. Fail. Anal..

[2] Araujo A.C., Mello G.M., Cardoso F.G. (2015). Thread milling as a manufacturing process for API threaded connection: Geometrical and cutting force analysis. J. Manuf. Process..

[3] Liu C.Y., Chang L.J. (2014). Out-of plane light-scattering polarimetric imaging of a thread surface. Opt. Laser Eng..

[4] Tong Q.B., Jiao C.Q., Huang H., Li G.B., Ding Z.L., Yuan F. (2014). An automatic measuring method and system using laser triangulation scanning for the parameters of a screw thread. Meas. Sci. Technol..

[5] Huang H.L., Jywe W.Y., Liu C.H., Duan L., Wang M.S. (2010). Development of a novel laser-based measuring system for the thread profile of ballscrew. Opt. Laser Eng..

[6] Mutilba U., Gomez-Acedo E., Kortaberria G., Olarra A., Fabra Y. (2017). Traceability of on-machine tool measurement: A review. Sensors.

[7] Li X.Q., Wang Z., Fu L.H. (2016). A laser-based measuring system for online quality control of car engine block. Sensors.

[8] Li X.Q., Wang Z., Fu L.H. (2016). A fast and in-situ measuring method using laser triangulation sensors for parameters of the connecting rod. Sensors.

[9] Wang L., Yang F.Y., Fu L.H., Wang Z., Yang T.Y., Liu C.J. (2017). A fast measuring method for the inner diameter of coaxial holes. Sensors.

[10] Zhang F.M., Qu X.H., Ouyang J.F. (2012). An automated inner dimensional measurement system based on a laser displacement sensor for long-stepped pipes. Sensors.

[11] Chen Y.Y., Chang R.S., Jwo K.W., Hsu C.C., Tsao C.P. (2015). A non-contact pulse automatic positioning measurement system for traditional Chinese medicine. Sensors.

[12] Korosec M., Duhovnic J., Vukasinovic N. (2010). Identification and optimization of key process parameters in noncontact laser scanning for reverse engineering. Comput.-Aided Des..

[13] Alam A., O’nils M., Manuilskiy A., Thim J., Westerlind C. (2015). Limitation of a line-of-light online paper surface measurement system. IEEE Sens. J..

[14] Mueller T., Poesch A., Reithmeier E. (2015). Measurement uncertainty of microscopic laser triangulation on technical surfaces. Microsc. Microanal..

[15] Rico J.C., Valino G., Fernandez P., Zapico P., Blanco D. (2015). Adjustment recommendations of conoscopic holography sensor for a reliable scanning of surfaces with roughness grades obtained by different processes. Precis. Eng..

[16] Yang H.W., Tao W., Zhang Z.Q., Zhao S.W., Yin X.Q., Zhao H. (2017). Reduction of the influence of laser beam directional dithering in a laser triangulation displacement probe. Sensors.

[17] Lee R.T., Shiou F.J. (2011). Multi-beam laser probe for measuring position and orientation of freeform surface. Measurement.

[18] Sun B., Li B. (2015). A rapid method to achieve aero-engine blade form detection. Sensors.

[19] Chen J.L., Wan Z.G., Pan J., Zi Y.Y., Wang Y. (2016). Customized maximal-overlap multiwavelet denoising with data-driven group threshold for condition monitoring of rolling mill drivetrain. Mech. Syst. Signal Process..

[20] Zhang W., Quan W., Guo L. (2012). Blurred star image processing for star sensors under dynamic conditions. Sensors.

[21] Donoho L.D., Johnstone I.M. (1994). Ideal spatial adaptation by wavelet shinkage. Biometrika.

[22] Nasri M., Nezamabadi-pour H. (2009). Image denoising in the wavelet domain using a new adaptive thresholding function. Neurocomputing.

[23] Yi T.H., Li H.N., Zhao X.Y. (2012). Noise smoothing for structural vibration test signals using an improved wavelet thresholding technique. Sensors.

[24] Gong T.R., Yang Z.J., Wang G.S., Jiao P. (2017). Supervised and unsurpervised subband adaptive denoising frameworks with polynomial threshold function. Math. Probl. Eng..

[25] Zhang Q., Wang L., Gao P.Y., Liu Z.J. (2016). An innovative wavelet threshold denoising method for environmental drift of fiber optic gyro. Math. Probl. Eng..

[26] Lu J.Y., Lin H., Ye D., Zhang Y.S. (2016). A new wavelet threshold function and denoising application. Math. Probl. Eng..

[27] General Administration of Quality Supervision, Inspection and Quarantine of the People’s Republic of China, Standardization Administration of the People’s Republic of China (2008). GB/T 22512-2008 Petroleum and Natural Gas Industries-Rotary Drilling Equipment.

[28] Jia T.Y., Wang H.Y., Shen X.H., Jiang Z., He K. (2018). Target localization based on structured total least squares with hybrid TDOA-AOA measurements. Signal Process..

